# Circulating Semaphorin 4D as a Marker for Predicting Radiographic Progression in Patients with Rheumatoid Arthritis

**DOI:** 10.1155/2018/2318386

**Published:** 2018-11-14

**Authors:** You-Jung Ha, Dong Woo Han, Ji Hyoun Kim, Sang Wan Chung, Eun Ha Kang, Yeong Wook Song, Yun Jong Lee

**Affiliations:** ^1^Division of Rheumatology, Department of Internal Medicine, Seoul National University Bundang Hospital, Seongnam, Republic of Korea; ^2^Division of Rheumatology, Department of Internal Medicine, Chungbuk National University Hospital, Cheongju, Republic of Korea; ^3^Division of Rheumatology, Department of Internal Medicine, Kyung Hee University Medical Center, Seoul, Republic of Korea; ^4^WCU Department of Molecular Medicine and Biopharmaceutical Sciences, Medical Research Institute, Seoul National University College of Medicine, Seoul, Republic of Korea; ^5^Department of Internal Medicine, Seoul National University Hospital, Seoul, Republic of Korea; ^6^Department of Translational Medicine, College of Medicine, Seoul National University, Seoul, Republic of Korea

## Abstract

Semaphorin 3A (Sema3A) and semaphorin 4D (Sema4D) are molecules which regulate immune responses as well as bone remodeling process. The aim of this study was to evaluate the serum levels of Sema3A and Sema4D and to investigate their clinical significance in rheumatoid arthritis (RA). The serum levels of Sema3A and Sema4D were measured in 130 patients with RA and 65 sex- and age-matched healthy individuals. Circulating levels of biomarkers of RA-related inflammation and bone turnover such as tumor necrosis factor- (TNF-) *α*, interleukin- (IL-) 6, IL-22, IL-34, osteopontin, Dkk-1, and sclerostin were also measured. Disease activity was determined by the 28-joint disease activity score (DAS28), and radiographic joint damage was assessed by the modified Sharp van der Heijde score (SHS). The serum levels of Sema3A were significantly higher in patients with RA than those in healthy controls (*p* < 0.001), whereas serum4D levels did not differ between the two groups. The levels of Sema4D showed a positive correlation with C-reactive protein (*p* = 0.001) and IL-6 (*p* < 0.001) levels, whereas the levels of Sema3A showed a negative correlation with Dkk-1 (*p* = 0.007) and TNF-*α* (*p* = 0.001). Even though Sema3A and Sema4D levels were comparable between RA patients with DAS28> 3.2 and with DAS28 ≤ 3.2, RA patients with radiographic progression (ΔSHS change/year ≥ 1) had significantly higher baseline levels of Sema4D than those without progression (*p* = 0.029). Additionally, when RA patients were divided into 3 groups using tertiles of Sema4D levels, the percentage of progressors was significantly increased (*p* = 0.045). In multivariate logistic regression analysis, serum Sema4D levels were an independent risk factor for radiographic progression. Our results suggest that the baseline levels of Sema4D might be a useful marker to identify RA patients with subsequent radiographic progression and that Sema4D may be an active mediator involved in RA-induced joint damage.

## 1. Introduction

Rheumatoid arthritis (RA) is a chronic systemic inflammatory arthritis characterized by synovitis of peripheral joints, which potentially results in irreversible joint destruction and disability. It is thought that the breakdown of immune tolerance triggered by environmental stimuli in genetically susceptible individuals leads to synovial inflammation and hypertrophy, pannus formation, neoangiogenesis, and subsequent degradation of adjacent cartilages and bones in RA [1]. Although the early diagnosis and intensive treatment of RA and the development of biologic disease-modifying antirheumatic drugs (DMARDs) have improved treatment outcomes [2–4], radiographic damage still occurs in a considerable number of RA patients. Approximately 20% of very early RA patients show erosive joint damage within 2 years, and progressive joint damage is also observed even in some RA patients with clinical remission as well as drug-free remission [5, 6]. Because the progression of joint damage is closely linked to disability in RA [7], a number of studies have attempted to identify prognostic markers for radiographic progression. For instance, RA patients positive for rheumatoid factor (RF) or anticitrullinated peptide antibodies (ACPA) have a greater risk of radiographic progression than those who are negative [8]. Additionally, several candidate biomarkers, including inflammatory proteins, cytokines, chemokines, and matrix-degrading enzymes, have been suggested in previous studies [8].

Semaphorins are a protein family containing a Sema domain of ~500 amino acids and have been initially identified as neural guidance molecules [9]. To date, more than 20 semaphorins have been found and are categorized into 7 classes, semaphorin 1 to 7 [10]. They are involved in various biological processes such as neuronal guidance, angiogenesis, regulation of tumor microenvironment, and immune cell responses. Several semaphorins are known as “immune semaphorins” because they are implicated in immune responses. For example, semaphorin 4D (Sema4D, also known as CD100) was the first protein to be identified as an immune semaphorin [11] and is involved in promoting B cell proliferation and T cell-dendritic cell (DC) cross-interaction [12, 13]. Semaphorin 3A (Sema3A) is another immune semaphorin, which can suppress T cell proliferation and promote the transmigration of DC across the lymphatics [14, 15]. Accordingly, the number of studies aimed at elucidating the roles of these immune semaphorins in autoimmune diseases has been increasing. Notably, soluble Sema4D levels have been found to be elevated in the serum and synovial fluid of patients with RA, and the administration of anti-Sema4D ameliorated inflammation in type II collagen-induced arthritis in mice [16]. Sema3A levels were lower in the serum and synovial fluid samples and synovial tissues of RA patients compared to those from osteoarthritis (OA) patients [17, 18]. Moreover, Sema3A overexpression attenuated collagen-induced arthritis [19].

Aside from their role in immune inflammation, recent studies have reported that Sema3A and Sema4D may also contribute to bone remodeling. Sema3A is secreted by osteoblasts and osteoclasts and exerts an osteoprotective effect by inhibiting receptor activator of nuclear factor-*κ*B ligand- (RANKL-) induced osteoclast differentiation [20]. Sema4D is highly expressed in osteoclasts and is cleaved into a soluble form upon osteoclast activation. It binds to plexin-B1 on osteoblasts and dose-dependently decreases bone formation through RhoA activation [21].

Joint inflammation precedes damage to the cartilage and bones in the early stage of RA, and chronic synovitis finally leads to bone erosion through the activation of osteoclasts and the suppression of osteoblasts. However, the association of semaphorins with joint damage in RA patients has not yet been evaluated. Based on the previously reported role of Sema3A and Sema4D, we hypothesized that these immune semaphorins are associated with an imbalance of bone remodeling in RA joints and could be potential biochemical markers of ongoing joint damage in RA patients. Hence, we measured the serum levels of Sema3A and Sema4D and investigated their clinical implications in radiographic progression in patients with RA.

## 2. Materials and Methods

### 2.1. Study Populations

One hundred thirty patients with RA and 65 sex- and age-matched healthy individuals were included in the study. All patients with RA fulfilled the 1987 revised American College of Rheumatology (ACR) criteria [22]. Serum samples were stored at −80°C until analysis. This study was approved by the Seoul National University Bundang Hospital's Institutional Review Board (IRB, B-0905/075-013) and was performed according to the recommendations of the Declaration of Helsinki. All the participants signed informed consent forms.

### 2.2. Clinical and Radiographic Assessment

Demographic and clinical data were collected at the time of blood sampling: age, disease duration, smoking status, and body mass index as well as medication use including conventional synthetic or biologic DMARDs, erythrocyte sedimentation rate (ESR, mm/h), and serum concentrations of C-reactive protein (CRP, mg/dL). RF and anticyclic citrullinated peptide (anti-CCP) antibody were measured using nephelometry with a cutoff of 15 IU/mL and enzyme-linked immunosorbent assay (ELISA) with a cutoff of 5 U/mL, respectively.

RA disease activity was assessed according to the 28-joint count disease activity score (DAS28) [23]. Patients with RA were divided into two subgroups according to their DAS28 scores: an active group, DAS28> 3.2, and an inactive group, DAS28 ≤ 3.2. Radiographs of both hands and feet were taken at baseline and repeated after a mean (±SD) 24.7 ± 15.5 months in all patients with RA. Radiographic damage was blindly assessed using the modified Sharp/van der Heijde score (SHS) by two trained investigators (YJH and SWC) [24]. The interobserver intraclass correlation coefficient (ICC) for individual SHS was 0.977 (95% confidence interval [CI], 0.971 to 0.982). ΔSHS ≥ 1 unit/year was defined as radiographic progression according to the previous literature [25].

### 2.3. Measurement of Semaphorins and Cytokines in Serum

The serum concentrations of Sema3A and Sema4D were determined using commercially available ELISA kits (MyBioSource, San Diego, CA, USA; Catalog No. MBS732622 for Sema3A and MBS2023012 for Sema4D) [26]. Assessment was performed according to the manufacturer's instructions, and the lower limits of detection were 0.156 ng/mL for Sema3A and 31.2 pg/mL for Sema4D. The levels of tumor necrosis factor- (TNF-) *α*, interleukin- (IL-) 6, IL-22, IL-23, osteopontin, Dickkopf-1 (Dkk-1), and sclerostin were analyzed with a Luminex 100 system (Luminex, Austin, TX, USA) using a magnetic bead-based immunoassay (R&D systems, Minneapolis, MN, USA). All measurements were performed in duplicate.

### 2.4. Statistical Analyses

The data were expressed as the median [interquartile ranges] or number (percentage) unless stated otherwise. For the comparisons between two groups, the Mann–Whitney test was used for continuous variable and Chi-square test or Fisher's exact test for categorical variables. The relationships among the continuous variables were determined using Spearman correlation coefficients. Interobserver reliability of SHS was assessed using the ICC. To ascertain semaphorins as an independent predictor of radiographic progression, binary logistic regression analysis was also performed and a model was constructed using covariates with a *p* value < 0.1 in the univariate analyses to compare patients with versus without radiographic progression and well-known risk factors such as active disease and seropositivity. Statistical analyses were performed using SPSS for Windows version 20 (IBM Corp, New York, USA). *P* values lower than 0.05 were considered statistically significant.

## 3. Results

### 3.1. Sema3A Levels Were Significantly Elevated in Patients with RA, but Not Sema4D

The baseline demographic, clinical, laboratory, and radiographic findings of participants are summarized in Table 1. The mean (±SD) age of RA patients was 52.9 ± 11.9 years old, and 111 patients (85.4%) were female. The median disease duration was 16 [4–81] months.

**Figure 1:**
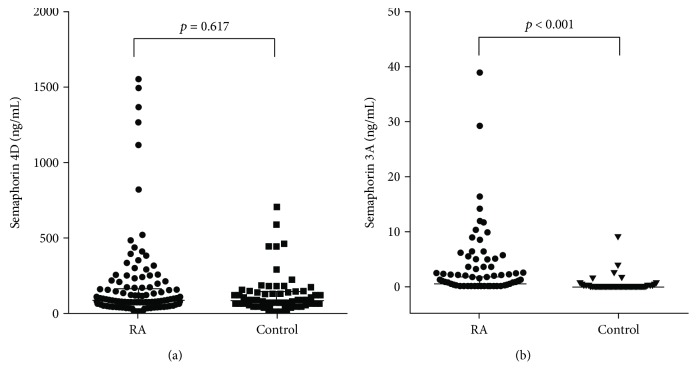
Serum levels of Sema4D (a) and Sema3A (b) in RA (*n* = 130) patients and controls (*n* = 65).

The serum concentrations of Sema3A and Sema4D in RA patients and in healthy subjects are shown in Figure 1. Whereas Sema4D levels were not different between RA patients and controls (88.3 [57.5–164.5] versus 91.1 [54.5–147.1] ng/mL, *p* = 0.617), Sema3A levels in RA patients were significantly higher than those in controls (0.44 [0–1.84] versus 0 [0–0.14], *p* < 0.001). The levels of IL-6, IL-23, and TNF-*α* in RA patients were significantly higher than those in the control group, but those of sclerostin were significantly lower. However, serum levels of Dkk-1 and osteopontin did not differ between the two groups (Table 2).

### 3.2. Associations of Clinical or Laboratory Features with Circulating Levels of Semaphorins in RA Patients

**Figure 2:**
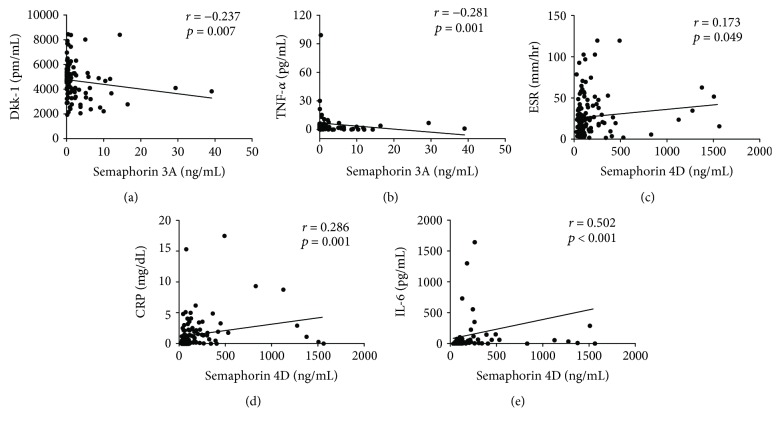
Associations of Sema3A and Sema4D levels with other biochemical data.

Sema3A levels showed negative correlations with Dkk-1 (*r* = −0.237, *p* = 0.007) or TNF-*α* (*r* = −0.173, *p* = 0.049) (Figures 2(a) and 2(b)). Sema4D levels were positively correlated with acute phase reactants (ESR and CRP) and serum IL-6 levels (*r* = 0.173 and *p* = 0.049 for ESR; *r* = 0.286 and *p* = 0.001 for CRP; *r* = 0.502 and *p* < 0.001 for IL-6; Figures 2(c)–2(e)). However, neither Sema4D nor Sema3A levels showed a significant correlation with baseline SHSs.

When the RA patients were divided into active (*n* = 82) and inactive (*n* = 43) subgroups according to their DAS28 status, the active subgroup showed a shorter disease duration, more DMARDs-naïve patients, and higher levels of ESR and CRP than the inactive subgroup (Supplementary Table S1). Additionally, the active subgroup showed significantly higher levels of IL-6 and osteopontin. However, there were no significant differences in the levels of Sema3A and Sema4D between these two subgroups.

### 3.3. Association of Elevated Levels of Sema4D in RA Patients with Radiographic Progression

**Figure 3:**
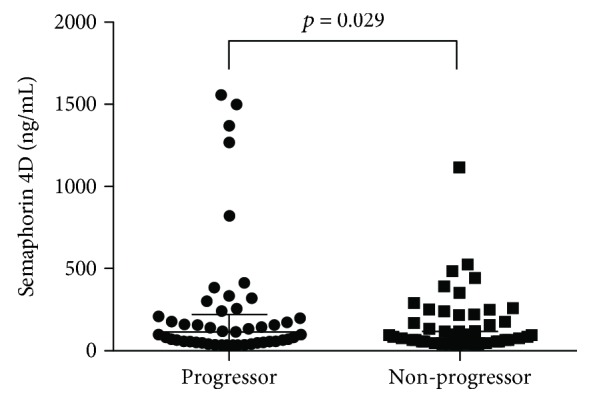
The relations of serum Sema4D levels with radiographic progression.

Among 135 patients with RA, 50 (37.0%) had radiographic progression over a median 21 months of follow-up. RA patients with radiographic progression were significantly older and had higher baseline scores of total SHS than those without progression (Table 3). The progressors were more likely to take hydroxychloroquine and tacrolimus at baseline. In addition, serum Sema4D levels were significantly higher in the radiographic progressors than those in the nonprogressor group (Figure 3). However, Sema3A levels were comparable between the progressors and the nonprogressors.

The proportion of progressors was significantly different across the tertiles of Sema4D levels (14/43 (32.6%) versus 13/44 (29.5%) versus 23/43 (53.5%), *p* = 0.045 by chi-square test). Furthermore, in the multivariate logistic regression analysis, serum Sema4D levels were an independent predictor for subsequent radiographic progression (odds ratio = 1.002 [95% CI, 1.000–1.003], *p* = 0.043; Table 4).

## 4. Discussion

In the present study, we evaluated the serum levels of the two immune semaphorins (Sema4D and Sema3A) and studied the association between their baseline levels and subsequent radiographic progression in patients with RA. We found that the serum levels of Sema3A were elevated in RA patients but were not related to radiographic progression. On the contrary, despite no difference in Sema4D levels between RA patients and controls, baseline Sema4D levels were significantly higher in those with radiographic progression than those without progression. Serum Sema4D concentrations were positively correlated with ESR, CRP, and IL-6 levels in RA patients, and baseline Sema4D levels remained a significant predictor for radiographic progression in the multivariate analysis. These findings suggest that Sema4D could be a novel biomarker predicting structural changes in the joints of patients with RA.

The cellular and molecular pathways of joint damage in RA patients include invasive pannus formation, enzymatic destruction of the extracellular matrix by proteases such as matrix metalloproteinases or ADAMTs, and increased osteoclastogenesis induced by RANKL and other cytokines produced by RA synovium [1, 27]. Proinflammatory cytokines such as TNF-*α*, IL-1*β*, and IL-17, in conjunction with IL-6 and tumor growth factor- (TGF-) *β*, stimulate fibroblast-like synoviocytes to synthesize RANKL and macrophage colony-stimulating factor (M-CSF), which augment osteoclast differentiation and activation [28, 29]. OPG (osteoprotegerin), a decoy receptor of RANKL, blocks binding of RANKL with its receptor RANK, resulting in the prevention of bone destruction [30]. In addition, the Wnt-mediated signaling pathway plays a crucial role in regulating synovial inflammation and bone remodeling [31]. Suppression of osteoblast function by the Wnt signaling inhibitors, including Dkk-1 and sclerostin, is involved in inflammatory bone loss along with OPG [32, 33].

Since joint damage in RA is irreversible and progressive, at times resulting in permanent disability depending on the extent of the damage, it is important to identify biomarkers that are predictive of joint damage in developing patient-tailored therapy. Previous studies have reported seropositivity for RF and/or ACPA as a risk factor to develop radiographic damages in RA patients [34, 35]. Elevated levels of cytokines such as IL-6, IL-22, IL-33, and IL-34, which are involved in the joint damage process, were previously reported to be related to radiographic progression [36–40]. Another study suggested high baseline levels of the RANKL : OPG ratio as a predictor of joint damage progression over the 11-year follow-up of RA patients [41]. Seror and his colleagues demonstrated that elevated levels of Dkk-1 were associated with radiographic progression even after adjustment of known predictive factors of joint damage (erosions at baseline and anti-CCP positivity) [42]. A single biomarker could not reflect all aspects of the complicated pathogenic pathways by which bone remodeling imbalance is facilitated in RA joints. Therefore, if we have more independent risk factors for radiographic progression, we can make a more accurate prediction.

In the present study, we also measured several previously known risk factors for radiographic progression. However, seropositivity for RF and/or ACPA, disease activity, and serum baseline levels of TNF-*α*, IL-6, IL-22, IL-23, osteopontin, Dickkopf-1 (Dkk-1), and sclerostin were not associated with subsequent joint damage. These results, which were inconsistent with previous studies, may be explained by differences in the study population (early RA versus late RA, treatment-naïve versus on treatment, and seropositive RA versus seronegative RA), the size of the study population, and the methods used for the assessment of radiographic damage (e.g., SHS vs. Larsen method) or biomarkers. For example, the prevalence of anti-CCP positive RA was 89% among our study participants but was 61% in Syversen et al.'s study [35]. In our study, old age was independently associated with radiographic progression. Previous studies have shown that RA joint damage constantly increased 1.034-fold per year increase in age and clinical predictors for erosion-free status over 2 years included a younger age at onset of RA [43, 44].

Sema4D-deficient mice showed immunological functional defects without apparent abnormalities in other tissues [45]. This immune semaphorin is constitutively expressed on T cells, is upregulated with T cell activation, and plays a role in T cell-mediated immune response through CD45 activation. Sema4D also exists in a soluble form generated by metalloproteinase-mediated proteolytic cleavage. Sema4D acts as a ligand for receptor plexins (B1, B2, and C1) and CD72 and has been reported to be involved in platelet and neutrophil activation, angiogenesis, and cancer metastasis [10, 46]. Additionally, Sema4D has been considered a major coupling factor for osteoclast and osteoblast in bone remodeling processes; osteoclast-derived Sema4D inhibits osteoblast differentiation [21]. With this biological background, Sema4D could play a pivotal role in osteoimmunology. However, the role of Sema4D in immune inflammation and joint destruction has been scarcely studied in RA, a typical disease falling into the category of osteoimmunology.

Yoshida et al. recently showed that Sema4D levels were significantly associated with several disease activity markers (e.g., DAS28 and CRP) in RA patients and found that Sema4D-stimulated CD14+ monocytes increased the secretion of TNF-*α* and IL-6 [16]. In the current study, serum Sema4D levels were also found to be significantly correlated with ESR, CRP, and IL-6 levels. Therefore, Sema4D levels could reflect the active T cell-mediated inflammatory process. Additionally, it is well known that IL-6 and its family members are upregulated in RA joints and, even in a RANK-deficient state, TNF-*α*/IL-6 can induce osteoclast formation and bone erosion [47]. Although Yoshida et al. did not find that serum Sema4D levels were cross-sectionally correlated with bone mineral density, we revealed that baseline Sema4D levels were associated with subsequent progression of radiographic damage after about 2 years in RA patients. Our results suggest that serum Sema4D level could be a biomarker associated with altered bone remodeling in RA joints. In the study by Yoshida and his colleagues, Sema4D levels were elevated in the serum and synovial fluid of RA patients. However, our study did not find higher circulating levels of Sema4D in RA patients. Yoshida et al.'s study did not provide detailed data on their controls consisting of staffs and students. In Yoshida et al.'s and our studies, commercial Sema4D ELISA kits from the same manufacturer were used, but since the ranges of serum Sema4D levels measured were very different between the two studies (mean ± SD 5.6 ± 3.1 versus 129.6 ± 132.6 ng/mL), it is possible that the kits could have been composed of different reagents. In fact, the serum levels of Sema4D were greatly heterogeneously reported in each study, from 0.58 ng/mL [48] to 762.2 ng/mL [49]. Therefore, the optimization and standardization of the measurement of Sema4D would be required for its clinical application.

Sema3A is another immune semaphorin that acts as a negative regulator of lymphocytic function in the pathogenesis of several autoimmune diseases, such as systemic lupus erythematosus and systemic sclerosis [10]. Sema3A has been reported to have an osteoprotective effect by enhancing osteoblastogenesis (via activation of activates Rac1 and canonical Wnt signaling) and suppressing osteoclastogenesis (via activation of PLC*γ* or inhibition of RhoA signaling) [50]. A low expression of Sema3A in the CD4+ T cells and synovial tissues of RA patients and an alleviation of collagen-induced arthritis by Sema3A overexpression have been previously reported [17, 19]. Additionally, Vadasz et al. found that the serum levels of Sema3A were significantly lower in RA patients compared with those in the controls [51]. On the contrary, several studies reported that serum Sema3A levels were rather elevated in patients with inflammatory diseases including RA, as confirmed by our own results [52–54].

These conflicting results on the levels of secreted Sema3A may be due to small sample sizes and heterogeneous RA patients in individual studies. Vadasz et al. enrolled only 24 RA patients and the study of *Gao* et al. and ours did 130 patients with RA [51, 52]. Also, measured expression levels of Sema3A could be different according to the sample types or detection methods. Sema3A expression levels in CD4+/CD25+ T cells were significantly lower in patients with inflammatory bowel diseases than in controls, although their serum Sema3A levels were significantly higher [26, 53]. Catalano reported that anti-CD3/CD28-stimulated peripheral blood mononuclear cells (PBMC) expressed lower levels of Sema3A protein in RA patients than in controls, but Gao et al. showed increased levels of its mRNA in RA PBMC [19, 52].

Moreover, we observed no significant association of Sema3A levels with baseline and subsequent radiographic damage. Although Sema3A-deficient mice showed a severe osteopenic phenotype [20], a human study did not demonstrate a significant association between serum Sema3A levels and bone biochemical markers or bone mineral densities in pre- and postmenopausal women [55]. Putting the above findings together, circulating Sema3A levels might not be an optimal biomarker for predicting joint damage in RA patients.

This study has several limitations. Firstly, the sample size was not large and we did not perform a sensitivity analysis. Further large-scale studies are needed to replicate our findings. Secondly, in the current study, longitudinal changes in the levels of immune semaphorins were not available and we could not examine whether their levels were significantly altered with antiarthritic treatments. Additionally, since approximately 60% of the RA participants were on conventional synthetic or biologic DMARDs at the time of enrollment, the use of these anti-inflammatory drugs may have altered their serum levels. However, we did not find these drugs to have an effect on neither Sema3A nor Sema4D levels. Finally, we did not obtain data on the bone mineral densities of the study participants and therefore did not study whether Sema4D had an effect on systemic bone mineral density. Nevertheless, our study is the first study to measure the levels of Sema3A, Sema4D, and cytokines and regulators related to inflammatory bone loss together and to determine the association of their levels with serial progression of radiographic damage.

## 5. Conclusions

In conclusion, this study suggests that serum Sema4D levels may be a new biomarker for predicting radiographic progression in patients with RA. Based on previous studies and our findings, targeting Sema4D can be a potential therapeutic option for RA in controlling inflammation as well as delaying radiographic damage.

## Figures and Tables

**Table 1:** Baseline characteristics of study population.

Characteristics	RA (*n* = 130)	Control (*n* = 65)	*p* value
Female sex	111	56	—
Age (years, mean ± standard deviation)	52.9 ± 11.9	52.7 ± 12.0	—
ESR (mm/hr)	20 (10–38)	5 (2–12)	<0.001
CRP (mg/dL)	0.67 (0.14–1.79)	0.03 (0.01–0.15)	<0.001
BMI (kg/m^2^)	22.5 [20.1–24.8]		
Disease duration (months)	16 [4–81]		
Current smoker	16/112 (14.3)		
66 swollen joint count	4 [1–9]		
68 tender joint count	3 [0–8]		
28 swollen joint count	2 [0–6]		
28 tender joint count	2 [0–5.3]		
DAS28-ESR	4.08 [2.88–5.09]		
RF positivity	102 (79.1)		
Anti-CCP positivity	112/126 (88.9)		
Comorbidities			
Osteoporosis	16 (12.3)		
Hypertension	22 (16.9)		
Diabetes mellitus	6 (4.6)		
Dyslipidemia	6 (4.6)		
Hypothyroidism	7 (5.4)		
Chronic hepatitis B	3 (2.3)		
Baseline modified SHS	3.5 [0–18]		
Erosion	1 [0–9]		
Joint space narrowing	2 [0–9]		
Duration between baseline and follow-up X-ray (months)	20.5 [13–27]		
Follow-up modified SHS	6 [1–23]		
Erosion	3 [0–13]		
Joint space narrowing	2 [0–11]		
SHS change/year	0.21 [0–1.95]		

Values are expressed as the median [IQR 25–75%] or *n* (%) unless stated otherwise. RA: rheumatoid arthritis; ESR: erythrocyte sedimentation rate; CRP: C-reactive protein; BMI: body mass index; DAS28: disease activity score 28; anti-CCP: anticyclic citrullinated peptide; SHS: Sharp/van der Heijde score.

**Table 2:** The levels of inflammatory cytokines and regulators of bone turnover between RA patients and healthy controls.

Characteristics	RA (*n* = 130)	Control (*n* = 65)	*p* value
TNF-*α* (pg/mL)	2.5 [0.5–3.9]	1.1 [0–2.8]	0.001
IL-6 (pg/mL)	7.6 [3.0–28.1]	0.6 [0–3.1]	<0.001
IL-22 (pg/mL)	0 [0–0]	0 [0–0]	0.316
IL-23 (pg/mL)	0 [0–8.7]	0 [0–0]	<0.001
Osteopontin (pg/mL)	5.8 [2.2–12.8]	6.0 [3.6–9.6]	0.742
Sclerostin (pg/mL)	26.2 [12.6–50.2]	31.8 [20.4–58.1]	0.001
Dkk-1 (pg/mL)	4665.7 [3846.0–5397.7]	4826.0 [3996.1–6086.6]	0.142

Values are expressed as the median [IQR 25–75%]. TNF-*α*: tumor necrosis factor-*α*; IL: interleukin; Dkk-1: Dickkopf-1.

**Table 3:** Comparisons of clinical and laboratory data between RA patients with and without radiographic progression.

Characteristics	Progressor (*n* = 50)	Nonprogressor (*n* = 80)	*p* value
Age (years)	56 [45–66.3]	51 [43–60]	0.036
Age > 60-year-old	22 (44.0)	19 (23.8)	0.016
Female	41 (82.0)	70 (87.5)	0.388
Body mass index (kg/m^2^)	23.2 [20.2–24.9]	22.0 [20.1–24.6]	0.578
Disease duration (months)	43.3 [5.0–115.1]	12.5 [2.2–75.2]	0.250
Current smoker	7 (17.9)	9 (12.3)	0.418
ESR (mm/hr)	22.5 [10–40.3]	18.5 [10–36.5]	0.535
CRP (mg/dL)	0.71 [0.14–2.03]	0.65 [0.13–1.45]	0.555
66 swollen joint count	4 [0.5–7.5]	4 [1–9]	0.928
68 tender joint count	3 [0–5]	4 [0–9]	0.485
DAS28-ESR	3.22 [4.08–5.19]	4.06 [2.60–5.06]	0.456
Active RA (DAS28-ESR > 3.2)	36 (73.5)	46 (60.5)	0.137
RF positivity	38 (76.0)	64 (81.0)	0.495
Anti-CCP positivity	41 (85.4)	71 (91.0)	0.331
DMARDs-naïve	34 (68.0)	43 (53.8)	0.108
Current RA medications			
Glucocorticoid	27 (54.0)	31 (38.8)	0.089
Methotrexate	22 (44.0)	36 (45.0)	0.911
Hydroxychloroquine	16 (32.0)	13 (16.3)	0.036
Sulfasalazine	4 (8.0)	6 (7.5)	0.583
Leflunomide	8 (16.0)	13 (16.3)	0.970
Cyclosporin A	3 (6.0)	1(1.3)	0.158
Tacrolimus	8 (16.0)	3 (3.8)	0.018
Semaphorin 4D (ng/mL)	115.6 [66.1–221.7]	80.4 [54.3–119.7]	0.029
Semaphorin 3A (ng/mL)	0.73 [0.16–2.29]	0.25 [0–1.65]	0.083
Dkk-1 (ng/mL)	4.706 [3.971–5.410]	4.591 [3.777–5.416]	0.787
IL-22 (pg/mL)	0 [0–0]	0 [0–0]	0.073
IL-23 (pg/mL)	0 [0–8.7]	0 [0–8.7]	0.393
IL-6 (pg/mL)	12.9 [3.3–50.4]	6.6 [2.9–18.2]	0.156
Osteopontin (pg/mL)	6.63 [2.90–13.33]	5.38 [2.00–12.10]	0.271
Sclerostin (pg/mL)	25.3 [12.6–46.7]	26.9 [12.6–53.4]	0.945
TNF-*α* (pg/mL)	1.89 [0.37–4.09]	2.75 [0.84–3.91]	0.451
Baseline modified SHS	8 [1–23.3]	1 [0–11.5]	0.003
Erosion	3 [0–12]	1 [0–5.8]	0.019
Joint space narrowing	6 [3–16.8]	0 [0–5]	0.002

Values are expressed as the median [IQR 25–75%] or *n* (%) unless stated otherwise. ESR: erythrocyte sedimentation rate; CRP: C-reactive protein; DAS28: disease activity score 28; RF: rheumatoid factor; anti-CCP: anticyclic citrullinated peptide; DMARDs: disease-modifying antirheumatic drugs; Dkk-1: Dickkopf-1; IL: interleukin; TNF-*α*: tumor necrosis factor-*α*; SHS: Sharp van der Heijde score.

**Table 4:** Multivariate logistic regression analysis for radiographic progression in RA patients.

Variables	Odds ratios	95% confidence interval	*p* values
Tacrolimus	4.757	0.912–24.808	0.064
Sema4D	1.002	1.000–1.003	0.043
Age > 60-year-old	2.352	1.029–5.374	0.043

The model included age > 60-year-old, use of hydroxychloroquine, use of tacrolimus, use of glucocorticoid, rheumatoid factor, anti-CCP, baseline total SHS, DAS28, ESR, CRP, IL-22, Sema3A, and Sema4D.

## Data Availability

The data used to support the findings of this study are available from the corresponding author upon request.

## Supplementary Materials

Supplementary Materials Supplementary Table S1: comparisons of clinical characteristics and measured markers between patients with active and inactive RA.
