# Supplementary material for: Circulating Semaphorin 4D as a Marker for Predicting Radiographic Progression in Patients with Rheumatoid Arthritis
Source: Dis Markers. 2018 Nov 14;2018:2318386. doi: 10.1155/2018/2318386 (PMC6261241; doi:10.1155/2018/2318386)
Supplement: Supplementary Materials — Supplementary Table S1: comparisons of clinical characteristics and measured markers between patients with active and inactive RA. [file 2318386.f1.docx]

**Supplementary Materials**

*Supplementary 1.* TABLE S1. Comparisons of clinical characteristics and measured markers between patients with active and inactive RA.

| Characteristics | Active (n = 82) | Inactive (n = 43) | *p*-value |
| --- | --- | --- | --- |
| Age (years) | 55 [44-65] | 51 [45-61] | 0.289 |
| Female | 70 (85.4) | 38 (88.4) | 0.433 |
| Body mass index (kg/m^2^) | 22.4 [20.0-24.8] | 22.7 [20.7-24.9] | 0.473 |
| Disease duration (months) | 11.0 [2.0-76.2] | 37.6 [5.9-116.8] | 0.038 |
| Current smoker | 13 (17.3) | 3 (8.8) | 0.194 |
| White blood cell (/mm^3^) | 7580 [6665-9770] | 6000 [5090-7100] | <0.001 |
| Hemoglobin (g/dL) | 12.5 [11.5-13.4] | 13.0 [12.5-13.6] | 0.010 |
| Platelet (×10^3^/mm^3^) | 300 [240-348] | 258 [232-300] | 0.014 |
| ESR (mm/hr) | 30 [19-50] | 10 [4-14] | <0.001 |
| CRP (mg/dL) | 1.16 [0.17-2.31] | 0.27 [0.10-0.80] | 0.001 |
| 66 swollen joint count | 6.0 [3.0-10.3] | 0 [0-2.8] | <0.001 |
| 68 tender joint count | 5.5 [3.0-10.5] | 1 [0-1.8] | <0.001 |
| DAS28-ESR | 4.78 [4.07-5.37] | 2.51 [1.91-3.00] | <0.001 |
| RF positivity | 65 (80.3) | 33 (76.7) | 0.648 |
| Anti-CCP positivity | 75 (92.6) | 34 (81.0) | 0.055 |
| DMARDs-naïve | 43 (52.4) | 8 (18.6) | <0.001 |
| Semaphorin 4D (ng/mL) | 100.7 [63.1-187.6] | 77.8 [56.0-104.3] | 0.121 |
| Semaphorin 3A (ng/mL) | 0.47 [0.02-2.29] | 0.35 [0-1.24] | 0.261 |
| Dkk-1 (ng/mL) | 4.605 [3.766-5.384] | 4.676 [3.966-5.570] | 0.789 |
| IL-22 (pg/mL) | 0 [0-0] | 0 [0-0] | 0.304 |
| IL-23 (pg/mL) | 0 [0-8.7] | 0 [0-8.7] | 0.970 |
| IL-6 (pg/mL) | 14.95 [4.29-49.29] | 3.56 [1.75-8.47] | <0.001 |
| Osteopontin (pg/mL) | 7.32 [3.82-15.34] | 3.04 [1.30-7.78] | 0.001 |
| Sclerostin (pg/mL) | 29.5 [11.8-58.4] | 23.0 [14.0-33.8] | 0.268 |
| TNF-α (pg/mL) | 2.34 [0.75-4.69] | 2.59 [0.49-3.61] | 0.402 |
| Baseline modified SHS | 4 [0-15] | 2 [0-23] | 0.803 |
| Erosion | 1 [0-8] | 1 [0-8] | 0.740 |
| Joint space narrowing | 2 [0-7] | 1 [0-10] | 0.802 |
| SHS change/year | 0.57 [0-2.34] | 0 [0-1.37] | 0.107 |
| Radiographic progressor  (SHS change/yr ≥1) | 36 (43.9) | 13 (30.2) | 0.097 |

ESR, erythrocyte sedimentation rate; CRP, C-reactive protein; DAS28, disease activity score 28; RF, rheumatoid factor; anti-CCP, anti-cyclic citrullinated peptide; DMARDs, disease-modifying anti-rheumatic drugs; Dkk-1, Dickkopf-1; IL, interleukin; TNF-α, tumor-necrosis factor-α; SHS, Sharp van der Heijde Score.
